# Targeting of non-coding RNAs encoded by novel *MYC* enhancers inhibits the proliferation of human hepatic carcinoma cells in vitro

**DOI:** 10.1038/s41598-022-04869-w

**Published:** 2022-01-17

**Authors:** Hae In Choi, Ga Yeong An, Eunyoung Yoo, Mina Baek, Jin Choul Chai, Bert Binas, Young Seek Lee, Kyoung Hwa Jung, Young Gyu Chai

**Affiliations:** 1 Department of Bionanotechnology, Hanyang University, Seoul, 04673 Republic of Korea; 2 Department of Molecular & Life Science, Hanyang University, Ansan, Gyeonggi-do 15588 Republic of Korea; 3 Institute of Natural Science and Technology, Hanyang University, Ansan, 15588 Republic of Korea; 4 College of Veterinary Medicine, Seoul National University, Seoul, 08826 Republic of Korea; 5 Convergence Technology Campus of Korea Polytechnic II, Incheon, 21417 Republic of Korea; 6 Department of Biopharmaceutical Systems, Gwangmyeong Convergence Technology Campus of Korea Polytechnic II, Gwangmyeong-si, Gyeonggi-do 14222 Republic of Korea

**Keywords:** Cancer, Cell biology, Molecular biology

## Abstract

The proto-oncogene *MYC* is important for development and cell growth, however, its abnormal regulation causes cancer. Recent studies identified distinct enhancers of *MYC* in various cancers, but any *MYC* enhancer(s) in hepatocellular carcinoma (HCC) remain(s) elusive. By analyzing H3K27ac enrichment and enhancer RNA (eRNA) expression in cultured HCC cells, we identified six putative *MYC* enhancer regions. Amongst these, two highly active enhancers, located ~ 800 kb downstream of the *MYC* gene, were identified by qRT-PCR and reporter assays. We functionally confirmed these enhancers by demonstrating a significantly reduced *MYC* expression and cell proliferation upon CRISPR/Cas9-based deletion and/or antisense oligonucleotide (ASO)-mediated inhibition. In conclusion, we identified potential *MYC* enhancers of HCC and propose that the associated eRNAs may be suitable targets for HCC treatment.

## Introduction

The transcription factor (TF) MYC is essential for various cellular processes, including cell growth, proliferation, differentiation, and apoptosis^1^. *MYC* tightly regulates the normal state, but dysregulation of *MYC* is prevalent in cancer. *MYC* expression is upregulated in 50–60% of all cancers^2,3^. Accordingly, studies on the enhancers of *MYC* were conducted, and it was found that their location is cancer-specific^4^. For example, the *MYC* enhancer is located approximately 0.7 Mb downstream of the gene in prostate cancer, 70 kb upstream in pancreatic cancer, and 1.9 Mb upstream in glioma^5–7^. However, studies on *MYC* enhancers in HCC have been lacking.

HCC is the most common type of primary liver cancer. It is prevalent globally and a leading cause of cancer-related death^8,9^. Significant genetic and epigenetic alterations exist in HCC. Their accumulation in key genes involved in cell survival, proliferation, apoptosis, and metastasis leads to carcinogenesis^10^. The dysregulation of *MYC* plays a vital role in proliferation and invasion, including tumor initiation and progression in HCC^11,12^.

Generally, both genetic and epigenetic changes could lead to altered *MYC* expression^13^. In the present study, we started with an epigenetic approach. Based on published genome maps featuring various epigenetic parameters, we identified novel *MYC* enhancers in HCC cell lines and then confirmed them with functional, genetic, and gene expression analyses. Specifically, we demonstrate that, at least in part, these novel enhancers operate through the expression of eRNAs.

eRNAs are noncoding transcripts generated by most, if not all, active enhancers and have been shown to play a central role in regulating gene expression^14^. They are often expressed cell type-specifically to control cell fate, and in cancer, they can be used as new diagnostic markers and drug targets^15,16^. The present study suggests that the same approach may also be applicable to HCC.

## Results

### Effects of BET inhibition on HCC cell proliferation and *MYC* expression

**Figure 1:**
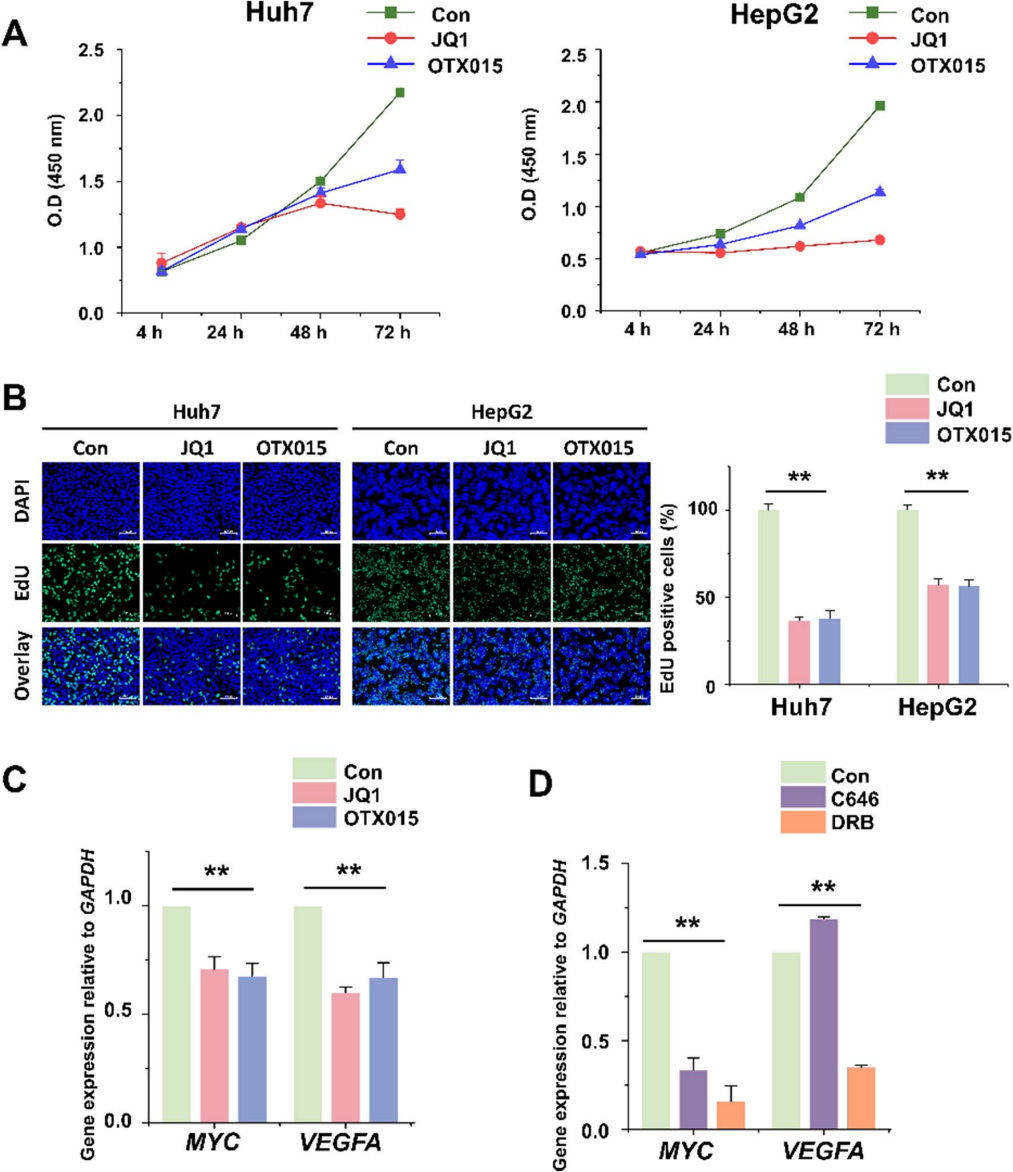
BET inhibitor suppresses cell viability, cell proliferation, *MYC* expression, and MYC target gene expression in Huh7 and HepG2 cells. (**A**) Cell viability was determined by the WST1 assay and represented by the relative absorbance at 450 nm. Huh7 and HepG2 cells were treated with 5 µM BET inhibitors (JQ1 or OTX015) for different durations (4 h, 24 h, 48 h, and 72 h). (**B**) The proliferation of Huh7 and HepG2 cells treated with 5 µM BET inhibitors for 24 h was assessed by EdU assay. Representative images and the number of EdU-positive cells (%) are shown. Original magnification, × 400. The data represent three biologically independent experiments. **p < 0.01. (**C**) qRT-PCR analysis of *MYC* mRNA and MYC target genes and *VEGFA* mRNA in BET inhibitor-treated HepG2 cells (5 µM, 24 h). (**D**) qRT-PCR analysis of *MYC* mRNA and MYC target genes, *VEGFA* mRNA in p300/CBP inhibitor, C646 or RNA polymerase II (RNAPII) transcription elongation inhibitor, DRB-treated HepG2 cells (50 µM, 6 h). The values are presented as the mean ± SD from triplicate well measurements. *p < 0.05 and **p < 0.01.

The drugs, JQ1 and OTX015, inhibit the Bromodomain and Extra-Terminal motif (BET) protein family member BRD4 that is required to maintain super-enhancer (SE) activation^17^ by interacting with TFs and chromatin remodeling proteins^18^. In addition, BET inhibitors interfere with BRD4-associated eRNA elongation^19^. Therefore, to study the effects of enhancer inhibition on HCC cells, we incubated the Huh7 and HepG2 cell lines for up to 72 h with JQ1 or OTX015. Compared to controls, the numbers of Huh7 and HepG2 cells were significantly reduced after 48 h and 24 h, respectively, of inhibitor treatment (Fig. 1A). In both cell lines, inhibition for 24 h significantly decreased the proportion of 5-ethynyl-2’-deoxyuridine (EdU)-incorporating cells, indicating reduced proliferation (Fig. 1B). Next, in the HepG2 cells, we determined the effect of the BET inhibitors on the mRNA levels of *MYC* and *VEGFA* (encoding vascular endothelial growth factor A). The expression of *VEGF* is elevated in various cancers, including HCC, and is one of the targets of BET inhibitors^20–22^. Each inhibitor reduced both mRNA levels to approximately 70% (Fig. 1C). Furthermore, we measured the expression of these genes in response to two transcription inhibitors, p300/cAMP response element-binding (CREB)-binding protein (CBP) inhibitor C646 (50 µM, 24 h) and RNAPII transcription elongation inhibitor 5,6-dichloro-1-β-d-ribofuranosylbenzimidazole (DRB) (50 µM, 24 h). C646 treatment significantly reduced the expression of *MYC* but not of *VEGFA*, while DRB significantly reduced the expression of both genes (Fig. 1D).

Thus, proliferation of the cultured HCC cells was inhibited by the BET inhibitors along with a reduced *MYC* and *VEGFA* expression. Furthermore, the effect of BET inhibitors on reducing *MYC* expression was closely related to RNA transcription inhibition.

### Identification of putative *MYC* enhancers in HCC cell lines

**Figure 2:**
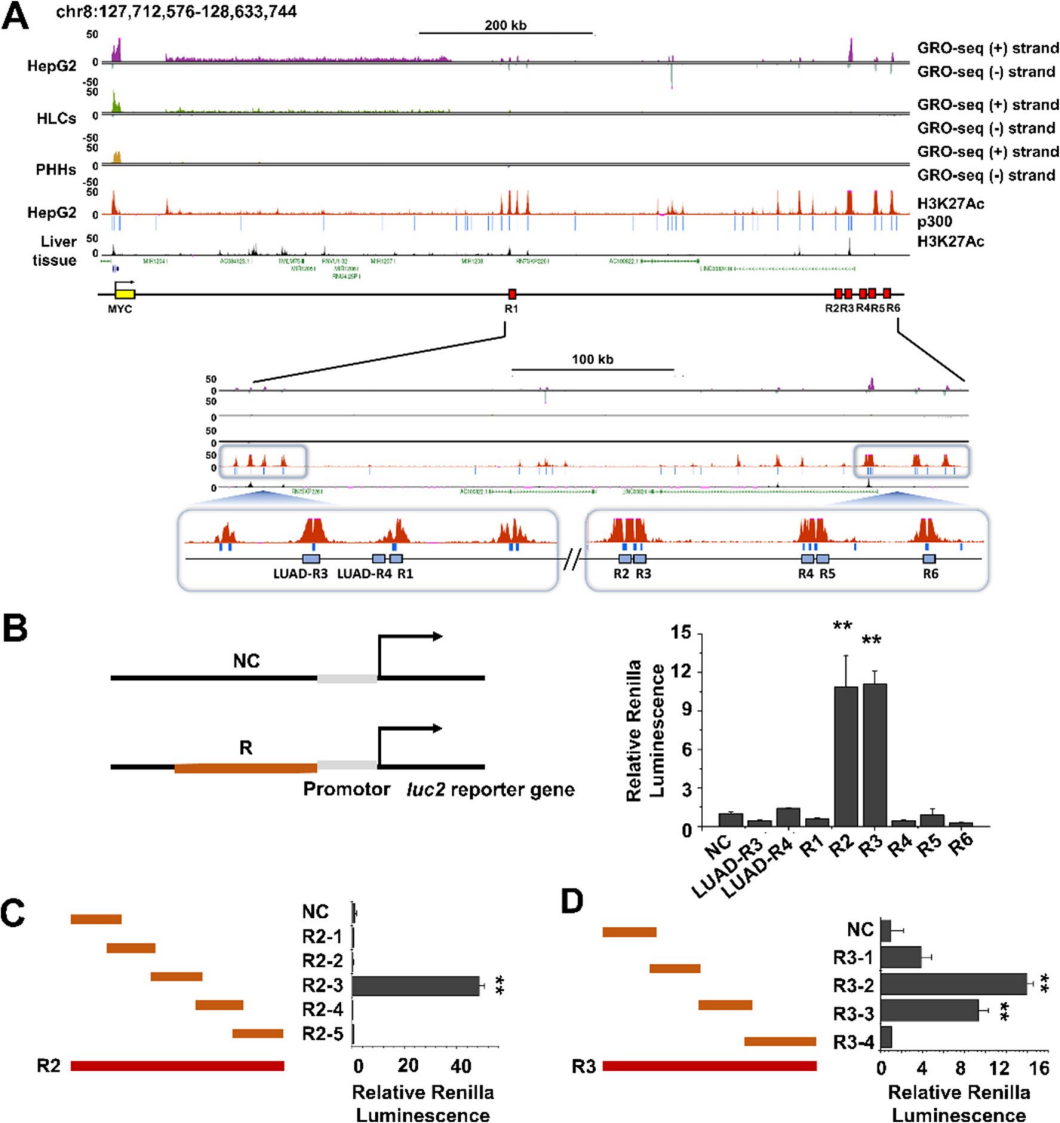
Identification of *MYC* enhancer in HepG2 cells. (**A**) USCS genome browser view of the GRO-seq peak, H3K27ac enrichment, and p300 binding sites along the *MYC* locus. Potential *MYC* enhancer is indicated + 500 kb downstream (red boxes). An enlarged display of H3K27ac-enriched reads aligned to the putative enhancer regions (GRCh38/Chr 8: 127,712,576–128,633,744). Six putative enhancer regions (R1, R2, R3, R4, R5, and R6) located downstream of the *MYC* gene are numbered. Hepatocyte-like cells, HLCs; primary human hepatocytes, PHHs; lung adenocarcinoma, LUAD. (**B**) Luciferase assay was used to identify the regions of active enhancers for *MYC* expression. These six putative enhancer regions were cloned upstream of the firefly luciferase reporter (*Luc2* gene). (**C**) The fragments of the R2 region were placed upstream of a luciferase reporter. (**D**) Fragments of the R3 region were placed upstream of a luciferase reporter. For each transfection, the firefly luciferase activity was normalized to that of the *Renilla reniformis* luciferase activity. The data are normalized to the pGL4.26 construct. The data represent three independent experiments. **p < 0.01.

Next, we examined ENCODE ChIP-seq and global run-on sequencing (GRO-seq) data of the HepG2 cell line in order to localize the potential *MYC* enhancers (Fig. 2). We found that the downstream regions of *MYC* were more enriched for H3K27ac, a histone mark for an active enhancer. Using the HepG2 cells GRO-seq peaks (GSE92375)^23,24^, H3K27ac ChIP-seq peaks (GSE29611)^25^, and p300 ChIP-seq (GSE32465)^26^ at the UCSC Genome Browser (http://genome.ucsc.edu), we identified six putative enhancer loci of the *MYC* gene (R1-6) (Fig. 2A, Supplementary Table S4). We did not observe the same peaks in iPSC-induced hepatocyte-like cells (GSM3271003), primary hepatocytes (GSM3271012)^27^, and liver tissue (GSM1112809)^28^ (Fig. 2A).

To seek direct functional evidence for the suspected enhancer activities in HCC cell lines, we examined the activity of a luciferase reporter in transiently transfected Huh7 cells. Each enhancer region was cloned into the minimal promoter vector pGL4.26 immediately upstream of the luciferase gene. For comparison, we included lung adenocarcinoma (LUAD)-R3 and LUAD-R4, two SE of *MYC* known to exhibit high and low activity, respectively, in lung adenocarcinoma cells^29^. As expected, transfection of R2 and R3 increased luciferase activity by approximately tenfold. In contrast, LUAD-R3 and LUAD-R4 did not show an enhanced activity (Fig. 2B). Next, we analyzed 500 bp fragments from within the R2 and R3 regions. Of the R2 fragments, the R2-3-containing plasmid showed the highest enhancer activity (Fig. 2C), while of the R3 fragments, R3-2- and R3-3-containing plasmid were most active (Fig. 2D). These results suggest R2 (R2-3) and R3 (R3-2 and R3-3) as candidate regulators of the transcriptional activation of *MYC* in HCC cells.

### eRNA of putative *MYC* enhancers in HCC cells

**Figure 3:**
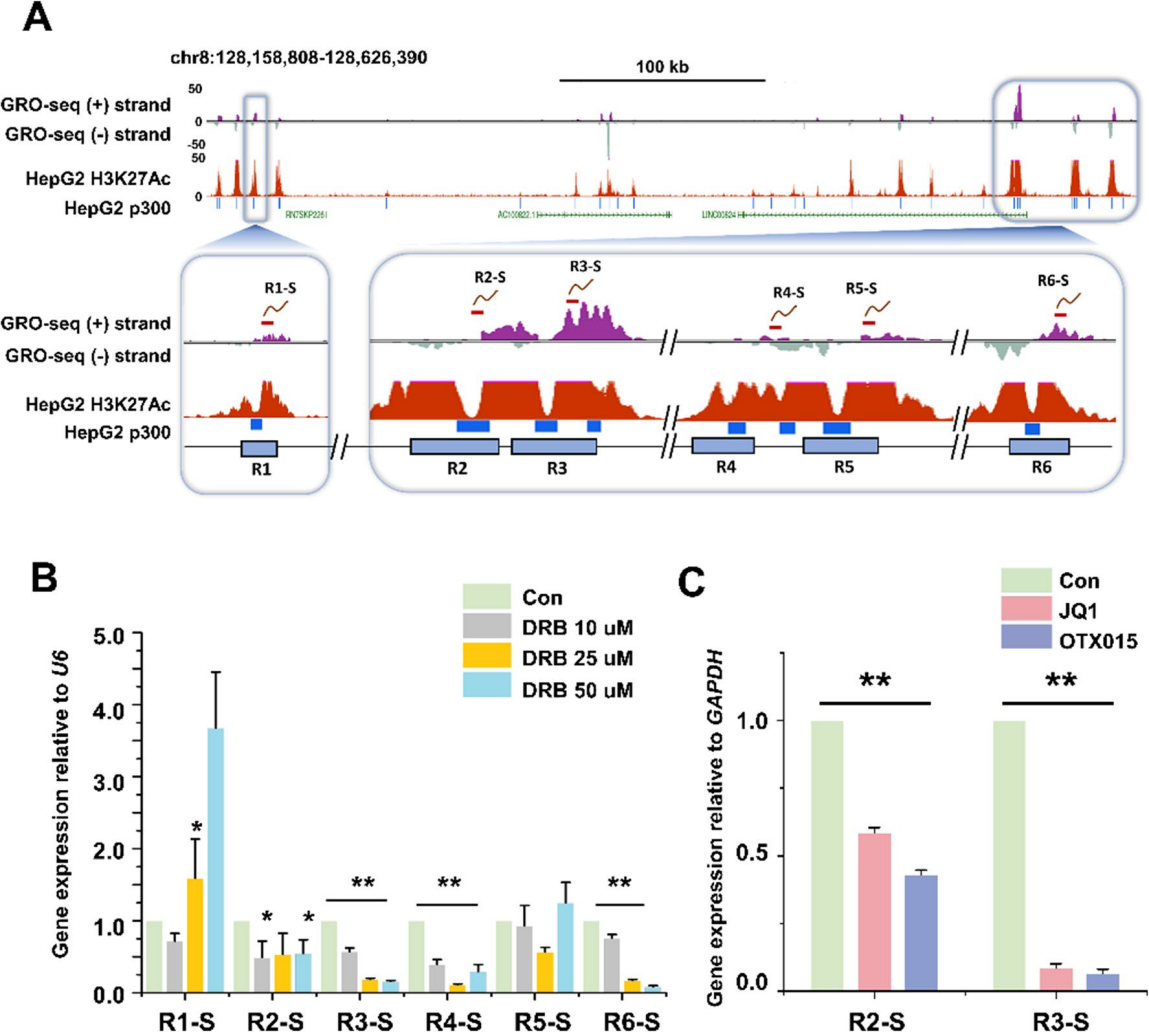
The eRNA expression by *MYC* enhancer regions in HepG2 cells. (**A**) USCS genome browser view of the GRO-seq peak, H3K27ac enrichment, and p300 binding sites along the *MYC* locus of HepG2 cells. An enlarged display of GRO-seq peak (+ strand and − strand) and H3K27ac enriched reads aligned to the putative enhancer regions (GRCh38/Chr 8: 128,158,808–128,626,390). eRNA primers were designed for six putative enhancer regions (R1, R2, R3, R4, R5, and R6). R1-S, R2-S, etc., stand for the sense strand eRNAs of the R1, R2, etc. regions. The red line above the GRO-seq (+) strand indicates target eRNA. (**B**) qRT-PCR of eRNA transcription levels in HepG2 cells treated with different concentrations of DRB (10 µM, 25 µM, and 50 µM; 6 h). (**C**) qRT-PCR of eRNA transcription levels in BET inhibitor-treated HepG2 cells (5 µM, 24 h). The values are the mean ± SD from triplicate well measurements. *p < 0.05 and **p < 0.01.

Next, we analyzed the R2 and R3 regions of the HepG2 cells for eRNA expression using qRT-PCR (Supplementary Table S1). Based on the GRO-seq data, six different sets of primers were designed (Fig. 3A). We found that the RNAPII transcription elongation inhibitor DRB caused a significant reduction in sense eRNA expression in regions R2, R3, R4, and R6 but not R1 and R5 (Fig. 3B). R2 and R3 were, therefore, further studied for expression changes through treatment with BET inhibitors. As expected, eRNA expression in regions R2 and R3 was significantly decreased (Fig. 3C). Additionally, R1, R4, R5, and R6 eRNAs were decreased in BET inhibitor-treated HCC cells (Fig. S1). Together, these results indicate that there is a correlation between the activity of enhancers and eRNA transcription. From this, we hypothesized that the BET inhibitors suppressed *MYC* expression through the regulation of eRNA expression.

### Disruption of *MYC* enhancers affects MYC-related gene expression and cell growth in HCC cells

**Figure 4:**
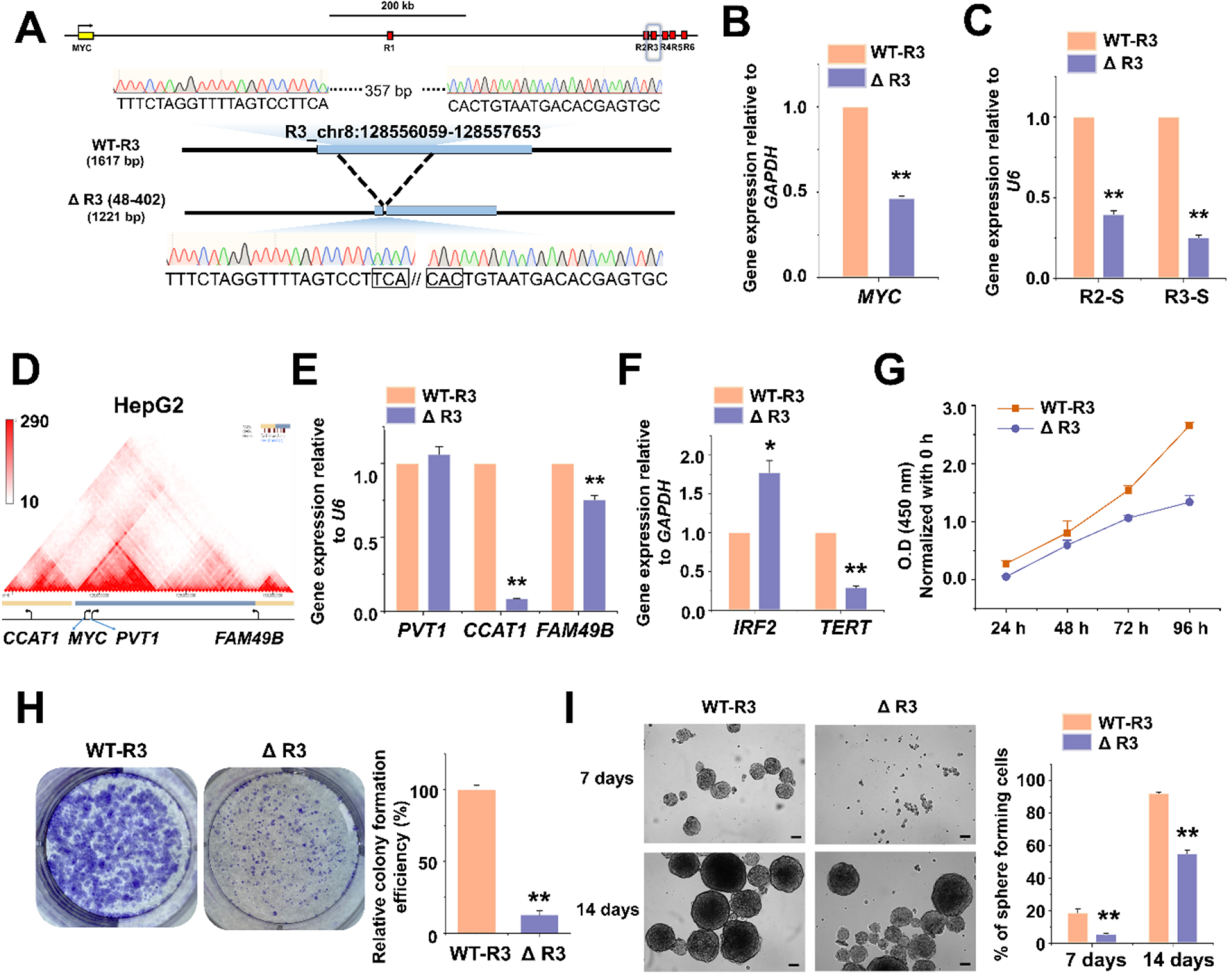
Generation of *MYC* enhancer region-deleted Huh7 cells via the CRISPR/Cas9 system. (**A**) Schematic illustration of the *MYC* enhancer locus structure and the wild-type (WT) and mutant (ΔR3) allele sequences around the R3 target region of Huh7 cells. (**B**) qRT-PCR results showing the levels of *MYC* mRNA, (C) R2 and R3-associated eRNAs in WT and R3-edited Huh7 cells. (**D**) TAD of *MYC* from Hi-C matrix of heatmap on chr8:126,840,000–130,080,000 in HepG2 cells (http://3dgenome.fsm.northwestern.edu/view.php). (**E**) qRT-PCR results showing the levels of *MYC*-related lncRNA and (**F**) *MYC*-related gene mRNAs in WT and R3-edited Huh7 cells. The values are the mean ± SD from triplicate well measurements. *p < 0.05 and **p < 0.01. (**G**) Cell proliferation was determined using a WST-1 assay and represented by the relative absorbance at 450 nm. WT and edited Huh7 cells were cultured in a growth medium for 96 h. The obtained absorbance was normalized to each 0 h absorbance. The data represent three biologically independent experiments. **p < 0.01. (**H**) Colony formation ability of WT and R3-edited Huh7 cells. Cells were grown for 10 days and stained with Crystal Violet. The relative colony formation efficiency was measured as a percentage of the area covered by the colonies. The data represent three biologically independent experiments. **p < 0.01. (**I**) WT and R3-edited Huh7 cells were cultured in cancer stem cell (CSC) growth media for a spheroid formation assay under ultralow adherence conditions. Cells grown for 7 days and 14 days in spheroid-forming conditions are shown in bright-field images taken with a 4X objective. The number of large spheres (over 100 µm) was counted; scale bar = 100 µm. The data represent three biologically independent experiments. **p < 0.01.

Since our eRNA expression experiments showed enhancer activity of R3, we tested whether its deletion affects *MYC* gene expression. We performed the deletion in the Huh7 cell line using the CRISPR/Cas9 system. The targeted sequences are located on chromosome 8 (Chr 8: 128,556,059–128,557,653) downstream of the *MYC* gene (Supplementary Table S3). After plating the transfected suspension at limiting dilution density, genomic PCR revealed a deletion of the R3 region in one of the wells showing growth. DNA sequencing revealed a 357 bp deletion on Chr 8:128,556,457–128,556,814 (Fig. 4A). Agarose gel electrophoresis confirmed the R3 region deletion (Fig. S2). Note that we did not rule out that due to either delayed Cas9 activity or contamination with wild type cells, the expanded gene-manipulated cultures (which showed a reduced growth rate) used for analysis were not genetically homogeneous. Hence, the effects of R3 deletion reported below are minimum estimates.

The R3-deleted cells showed reduced *MYC* gene expression relative to wild-type cells (Fig. 4B). Using qRT-PCR analysis, we found that R2 eRNA (R2-S) and R3 eRNA (R3-S) expression were significantly decreased (Fig. 4C), and so was the expression of lncRNA *CCAT1* (Chr 8: 127,207,382–127,219,268), a gene that is not part of the same TAD (Topologically associated domains) as *MYC* but is known to be regulated by MYC in HCC^30^. By contrast, the expression of the neighboring lncRNA gene, *PVT1,* which is in the same TAD as *MYC*, was unaffected (Chr 8: 127,795,799–128,101,256). Similarly, *FAM49B*, which is not in the same TAD, was scarcely affected (Chr 8: 129,841,470–130,016,672)^31^ (Fig. 4D,E). We then analyzed the expression of two more MYC-related genes, *IRF2* and *TERT*, which are known to be repressed and activated by MYC, respectively^32,33^. In agreement, we found that *IRF2* was upregulated in R3-deleted cells; however, *TERT* was downregulated (Fig. 4F).

Next, we performed cell proliferation and colony formation assays. The R3-deleted cells exhibited a decreased proliferation and colony formation ability (Fig. 4G,H). Similarly, the R3 deletion reduced the spheroid formation by Huh7 cells (Fig. 4I).

It is known that *MYC* deletion is incompatible with proliferation^34^. It was therefore not surprising that our attempts to delete R2 and R3 did not result in well growing homogeneous cell lines. In fact, we could not recover R2-deleted cell cultures, suggesting a strong positive effect of R2 on *MYC* expression (see also further below the section on ASO inhibition). In contrast, we obtained passagable cultures that showed at least a significant proportion of R3-deleted cells (Fig. S2). These cultures exhibited a reduction of both the expression of *MYC* (Fig. 4B) and of their proliferation (Fig. 4G).

These results confirm that enhancer deletion influences cancer cell growth by reducing *MYC* expression^35^.

### Inhibition of *MYC* eRNA causes an effect equivalent to *MYC* enhancer disruption

**Figure 5:**
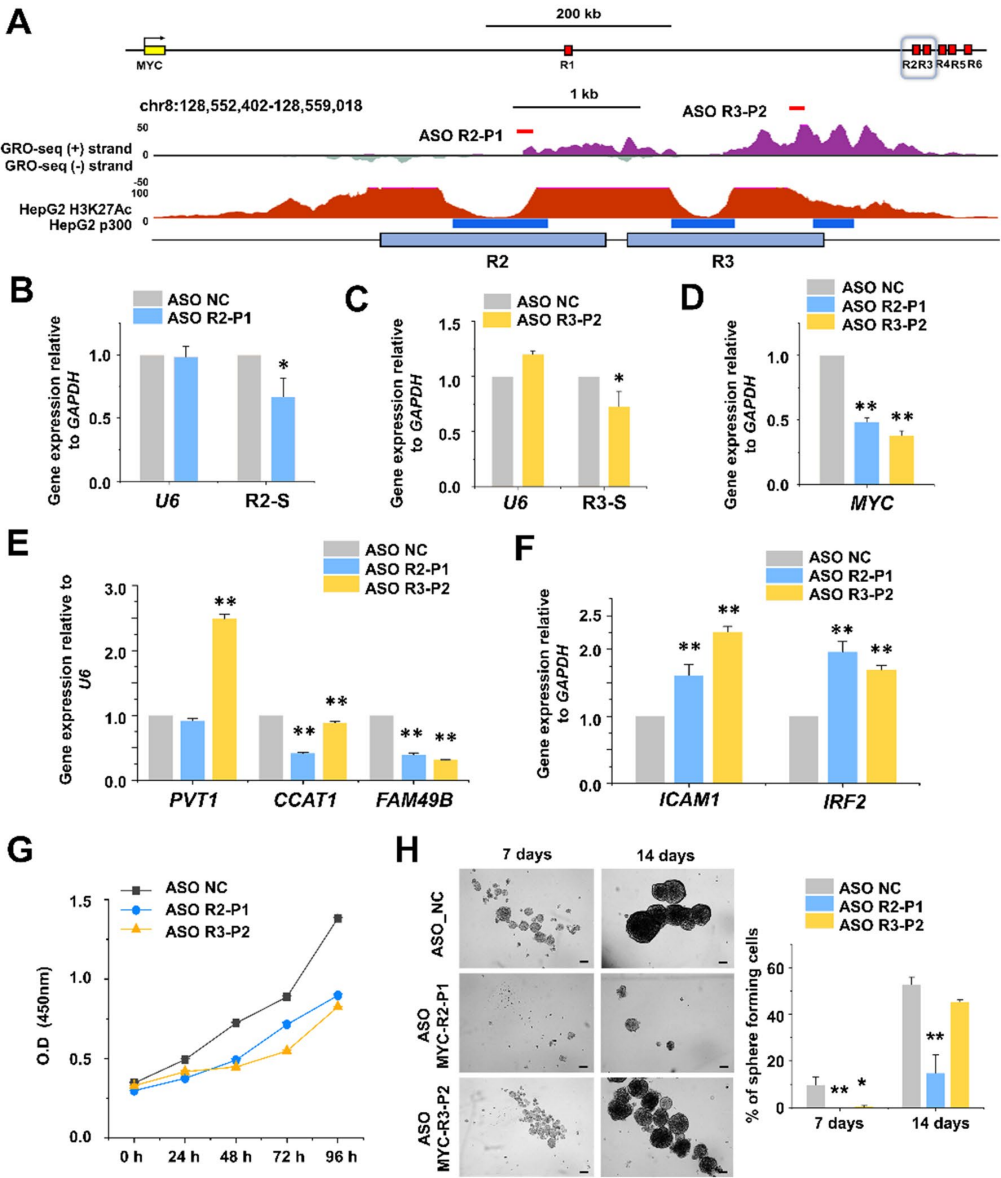
Effects of ASOs targeting *MYC* eRNAs in Huh7 cells. (**A**) Schematic illustration of the *MYC* enhancer locus structure and USCS genome browser view of the GRO-seq peak, H3K27ac enrichment, and p300 binding sites along the R2 and R3 regions. The targeting ASOs, ASO R2-P1 and ASO R3-P2, were designed to bind to the R2-S and R3-S eRNAs, respectively. (**B**,**C**) Huh7 cells were treated with 125 pmol of a non-targeting ASO (ASO NC) or the targeting ASO. Relative quantitation of R2-S (**B**) and R3-S (**C**) eRNA expression levels was performed by qRT-PCR. *U6* stands for the control gene *RNU6-1*. (**D**–**F**) Relative quantitation of *MYC* mRNA expression levels (**D**) and MYC-controlled mRNA expression levels (**E**,**F**). The values are the mean ± SD from triplicate well measurements. *p < 0.05 and **p < 0.01. (**G**) Cell proliferation was determined using a WST-1 assay and represented by the relative absorbance at 450 nm. Absorbance was measured every 24 h from 0 to 96 h after transfection of ASO. The data represent three biologically independent experiments. **p < 0.01. (**H**) ASO-transfected Huh7 cells were cultured in a growth medium for the spheroid formation assay. Cells grown for 7 days and 14 days in spheroid-forming conditions are shown in bright-field images taken with a 10X objective. The number of large spheres (over 100 µm) was counted; scale bar = 100 µm. The data represent three biologically independent experiments. **p < 0.01.

To further confirm the functional role of *MYC* eRNA, the antisense oligonucleotides, ASO R2-P1 and ASO R3-P2, were designed to bind the sense eRNAs at *MYC*-R2 (R2-S) and *MYC*-R3 (R3-S), respectively (Fig. 5A; Supplementary Table S1), and transfected into Huh7 cells. As expected, both eRNAs were specifically decreased by the corresponding ASOs (Fig. 5B,C). Importantly, both transfections also resulted in a significantly reduced *MYC* gene expression relative to the control (ASO NC) (Fig. 5D). Furthermore, when R2-S was targeted, the expression levels of the lncRNAs, *PVT1*, *CCAT1*, and *FAM49B*, were altered similarly as in the *MYC*-R3 deletion (Fig. 5E, compare with Fig. 4E). In addition, the expression of *IRF2* and *ICAM1,* another gene known to be repressed by MYC^36^, was increased by both ASOs (Fig. 5F, also compared with Fig. 4F).We next analyzed the proliferation and spheroid-forming abilities of the ASO-transfected Huh7 cells. Both ASO R2-P1 and ASO R3-P2 significantly reduced cell proliferation compared to ASO NC (Fig. 5G), again mimicking the R3 deletion (compare with Fig. 4G). In addition, the spheroid-forming assay showed that inhibition of R2- and R3-induced eRNAs by ASO treatment negatively affected growth (Fig. 5H). These results indicate that inhibition of eRNA can mimic a direct deletion of the corresponding chromosomal DNA (compare with Fig. 4).

## Discussion

The enhancer is a DNA region in which the E1A-binding proteins p300 (p300), RNAPII, and TFs are enriched through increased accessibility caused by histone modification. Enhancers help transcriptional activation directly through looping or indirectly affect transcription by expressing eRNAs^37,38^. Previous studies have revealed the presence of cancer-specific enhancers, the expression of eRNA in cancer cells, and the associated abnormal expression of oncogenes^39,40^. Since lineage-specific TFs affect the tumorigenesis function of their enhancers, they can also be cancer type-specific^41^. Therefore, we used ChIP-seq data from HepG2 cells as well as GRO-seq data that included nascent transcripts to identify eRNA expression, an active enhancer marker^42,43^. Based on these data, we found that the specific *MYC* enhancers R2 and R3 in HCC cell lines are significantly different from those in other cancers in terms of their location and activity^29,39,44^. In acute myeloid leukemia (AML), *MYC* expression is regulated by a SE that consists of five distinct small enhancers located 1.7 Mb downstream of the *MYC* promoter^40^. In colorectal cancer (CRC) and prostate cancer, the *MYC* enhancer is located 335 kb upstream of *MYC*^5^. In addition, the Zhang group deleted approximately 1.5 kb of the *MYC* enhancer located 450 kb downstream of the 3′ end in lung adenocarcinoma cells. *MYC* expression was reduced by 70%, and clonogenic growth was inhibited by approximately 50%. Note that we epigenetically identified the novel MYC enhancers in HepG2 cells (which are derived from a 15 years-old patient)^45^ but functionally confirmed these enhancers in Huh7 cells (which are derived from a 57 years-old patient)^46^. This coincidence strongly suggests the general significance of these newly found enhancers in hepatocyte tumorigenesis. It will be interesting to see whether the same enhancers may also be active in normal proliferating liver cells (such as in embryogenesis or liver regeneration).

eRNAs are generally upregulated in various cancers compared to normal tissues, and they can be used as pan-cancer diagnostic markers^47^. In addition, tissue-specific highly expressed eRNAs, such as *CCAT1* in colorectal cancer and androgen receptor (AR)-induced Kallikrein-related peptidase 3 (KLK3) eRNA (KLK3e) in prostate cancer, are considered new targets for treating various cancers^14,48,49^. Although the function of eRNAs has not been fully elucidated, eRNA depletion reduces the transcription of target genes by affecting alterations in chromatin structure and contributing to transcriptional initiation of target genes^49^. eRNA transcription can be regulated by inhibiting enhancer activity or effectively targeting ASO to control target gene expression and cancer cell progression^50,51^.

Previous studies have found that the inhibition of *MYC*-related and other eRNAs by using ASOs can effectively inhibit tumor cell progression, suggesting that eRNAs can be helpful as therapeutic targets^51–53^. For example, Epstein-Barr virus (EBV) super-enhancer (ESE) RNAs facilitated the expression of the *MYC* oncogene in lymphoma, and targeting ESE eRNA showed a therapeutic effect on EBV-related malignancies^53^. Similarly, in our results, targeting *MYC*-IEANC RNAs transcribed from *MYC* enhancers R2 and R3 in Huh7 cells caused an effective decrease in *MYC* expression along with reduction of proliferation and spheroid formation, suggesting a therapeutic effect on HCC. A previous study demonstrating that downregulation of MYC suppressed spheroid growth of colon CSCs and tumor growth in vivo^54^, lends support to our suggestion. Of note, the ASO-mediated inhibition of *MYC* eRNA expression may be superior compared to an alternative strategy, in which BRD4 inhibitors also suppressed tumor cell-associated *MYC* expression^5,55–57^. Unlike BET inhibitors, the selective targeting of *MYC* eRNAs probably avoids side effects such as toxicity and resistance.

## Conclusion

In this study, we identified the putative *MYC* enhancers of HCC cells. Enhancer activity and eRNA transcription were analyzed to determine the region involved in *MYC* expression, and it was found that both the deletion of the *MYC* enhancers and the ASO-mediated inhibition of the corresponding eRNAs suppressed the proliferation and reduced spheroid formation in HCC cell lines. Thus, our study suggests that for HCC, a strategy for reducing *MYC* expression through specific targeting with ASO has therapeutic potential without the side effects of gene editing or BRD4 inhibition. Future work will need to evaluate the role of the *MYC* enhancers identified here in HCC in vivo.

## Materials and methods

### Cell culture of HCCs

The HepG2 and Huh7 HCC cell lines were purchased from Korean Cell Line Bank (Seoul, Korea) and maintained in minimum essential medium or RPMI-1640 medium supplemented with 10% fetal bovine serum (FBS) and penicillin (100 units/ml)/streptomycin (100 μg/ml) (Thermo Fisher Scientific, Waltham, MA, USA). The medium was replaced every 3–4 days. The cells were maintained in a humidified incubator with 95% air and a 5% CO_2_ atmosphere at 37 °C. JQ1, OTX015, C646, and 5, 6-dichlorobenzimidazole 1-β-d-ribofuranoside (DRB) were purchased from Tocris Bioscience (Minneapolis, MN, USA). JQ1, OTX015, C646, and DRB were dissolved in dimethyl sulfoxide (DMSO) at a stock concentration of 10 mM. The cells were treated with different concentrations of JQ1 and DRB for different durations.

### Cell proliferation assays

Relative cell numbers were assessed using a premixed water-soluble tetrazolium salt (WST-1) cell viability test (Takara, Shiga, Japan) according to the manufacturer’s instructions. The cells were seeded at a density of 1 × 10^4^ cells per well. WST-1 was added to each well, and the absorbance of the microplate at 450 nm was measured after an additional 4 h incubation. The data represent three independent experiments (n = 3). DNA-synthesizing cells were visualized using an Ethynyl deoxyuridine (EdU) kit (Invitrogen, CA, USA) following the manufacturer’s instructions. Then, the cells were washed with phosphate-buffered saline, mounted with a 4’, 6-diamidino-2-phenylindole (DAPI)-containing mounting solution (Vectashield, Vector Laboratories, Burlingame, CA, USA), and imaged by microscopy (Nikon Eclipse 80i, Tokyo, Japan). The percentage of EdU-positive cells was examined in HCC cell lines using ImageJ (Bethesda, MD, USA) software. The data represent three independent experiments (n = 3).

### Gene expression analysis using quantitative PCR (qRT-PCR)

Total RNA was extracted from the cells using RNAiso Plus (Takara, Shiga, Japan) according to the manufacturer’s instructions. cDNA was synthesized by PrimeScript reverse transcriptase (Takara, Shiga, Japan) and amplified using gene-specific primers. The primers used for qRT-PCR are listed in Supplementary Table S1. The primers were designed by Primer Bank (https://pga.mgh.harvard.edu/primerbank/). qRT-PCR was performed with TBGreen *Premix Ex Taq* II (Takara, Shiga, Japan). Glyceraldehyde-3-phosphate dehydrogenase (*GAPDH*) was used as an internal control. The data represent three independent experiments (n = 3). After performing qRT-PCR, the results were analyzed using the critical threshold (ΔC_T_) and the comparative critical threshold (ΔΔC_T_) methods in ABI 7500 (Applied Biosystems, Foster City, CA, USA) software with the NormFinder and geNorm PLUS algorithms.

### Genomic data analysis

We re-analyzed two public ChIP-seq data sets in Gene Expression Omnibus (GEO) (GSE29611 and GSM1112809) according to the procedure described previously^58^, and three GRO-seq data sets in GEO (GSE92375, GSM3271003, and GSM3271012). For our re-analysis, the raw data were trimmed with Trimmomatic (version 0.36)^59^ and processed using Bowtie2 (version 2.3.5)^60^ or STAR (version 2.7.8)^61^ aligner software with a UCSC hg 38 reference. The ChIP-seq and GRO-seq peaks identified were analyzed with Homer (version 4.11)^62^ and visualized using UCSC Genome Browser (https://www.genome.ucsc.edu).

### Luciferase reporter assay

Enhancer regions (R1 ~ R6) were amplified using LongAmp *Taq* 2X Master Mix (New England Biolabs, Ipswich, MA, USA). Enhancer regions were amplified using forward and reverse primers to generate NheI or SacI and XhoI sites, respectively. These constructs were cloned into the pGL4.26 construct (Promega, Madison, WI, USA). The primers used for cloning are listed in Supplementary Table S2. The cells were seeded into 24-well plates and transfected with Lipofectamine 3000 (Thermo Fisher Scientific, Waltham, MA, USA). According to the manufacturer’s instructions, luciferase activity was measured using the Dual-Glo Luciferase Assay kit (Promega, Madison, WI, USA). PRL-TK (Renilla luciferase expression construct; Promega, Madison, WI, USA) was used as an internal control. Luciferase activity was normalized to Renilla luciferase and the control (empty vector).

### Construction of R3 region KO HCCs using the CRISPR/Cas9 system

Forward and reverse oligomers for gRNA synthesis against target sites were designed according to the manufacturer’s instructions. The oligomers were extended into 100-mer insert DNA using Phusion High-Fidelity PCR Master Mix (M0531, New England BioLabs, Ipswich, MA, USA) with the following setup: 2 min at 98 °C, 4 cycles of amplification (10 s at 98 °C, 20 s at 53 °C, 30 s at 72 °C), and 5 min at 72 °C. Then, the insert DNA was purified and combined with gRNA_Cloning Vector using Gibson Assembly Master Mix (E2611, New England Biolabs, Ipswich, MA, USA) at 50 °C for 1 h, followed by transformation and colony PCR. The cloned vectors were then purified and ordered to be sequenced (Macrogen, Seoul, Korea) to confirm the recombination. HCCs were transfected with the recombinant gRNA plasmid vector and hCas9 in a 95:5 ratio using Lipofectamine 3000 (L3000-001, Life Technologies, Carlsbad, CA, USA) according to the manufacturer’s instructions. The gRNA cloning vector and hCas9 was a gift from George Church^63^. The transfected cells were seeded into 96-well plates at a ratio of less than 1 cell per well. After ~ 2 weeks, cells from wells that showed growth were moved into 24-well plates. After further growth, a portion of each well was used to extract genomic DNA (gDNA) using a Wizard Genomic DNA Purification Kit (A1125, Promega, Madison, WI, USA). Then, gDNA was amplified by PCR with target-specific primers and sequenced to check properly generated deletions. The cells of one confirmed well were expanded in 100 mm dishes and then used for the various gene expression and proliferation assays. These analyses were performed within the first 10 passages.

### Knockdown of eRNA using ASO

Locked nucleic acid (LNA)-modified ASOs complementary to eRNA of MYC were designed from Antisense LNA GapmeRs (Qiagen, Hilden, Germany). The ASOs were purchased from Qiagen. The sequences are listed in Supplementary Table S3. For the transfection of Huh7 cells, ASOs were mixed with RNAiMAX in serum-free Opti-MEM (Gibco, Waltham, MA, USA). At varying concentrations of ASOs, dissolved Opti-MEM was added, and the cells were incubated in a growth medium for 4 h at 37 °C and 5% CO_2_. For total RNA extraction, the cells were harvested 48 h posttransfection.

### Colony formation assay

R3-deleted and WT Huh7 cells were seeded on 6-well plates in growth media at a density of 2500 cells/well and incubated in a CO_2_ incubator for 10 days. Then, the cells were washed with PBS, fixed with 4% paraformaldehyde for 20 min, and washed once with PBS. The cells were stained with 1% Crystal Violet (Sigma, St. Louis, MO, USA) for 30 min. After Crystal Violet was removed, the plates were washed with DW for 5 min and dried. The stained cells were analyzed for colony formation rates using ImageJ (Bethesda, MD, UAS).

### Spheroid formation assay

Cells were seeded on 24-well ultralow attachment culture dishes in growth media at a density of 1000 cells/well. DMEM/F12 serum-free medium (Gibco, Waltham, MA, USA) contained 2 mM l-glutamine, 1% sodium pyruvate (Invitrogen, Carlsbad, CA, USA), 100 U/ml penicillin, and 100 µg/ml streptomycin supplemented with 20 ng/ml epithelial growth factor (Invitrogen, Carlsbad, CA, USA), 10 ng/ml fibroblast growth factor-2 (Invitrogen, Carlsbad, CA, USA), N2 (R&D Systems, Minneapolis, MN, USA), and B27 (Invitrogen, Carlsbad, CA, USA). The cells were incubated in a CO_2_ incubator for one to two weeks, and oncosphere cells over 100 µm were counted with JuLI Br (NanoEnTek, Seoul, Korea).

### Statistical analysis

The data are presented as the mean ± standard deviation (SD) of the mean. All statistical analyses were performed using the IBM SPSS Statistics 26.0 program (IBM). We used a one-way analysis of variance followed by Tukey’s honestly significant difference post hoc test.* p* values < 0.05 were considered significant.

## Supplementary Information

Supplementary Information.

## Abbreviations

HCC: Hepatocellular carcinoma
eRNA: Enhancer RNA
ASO: Antisense oligonucleotide
SE: Super-enhancer
GRO-seq: Global run-on sequencing
H2K27ac: Acetylated H3 lysine 27
Chr: Chromosome
TAD: Topologically associated domains
CSC: Cancer stem cell
qRT-PCR: Quantitative reverse transcription-polymerase chain reaction

## References

[1] Pelengaris S, Khan M, Evan G (2002). c-MYC: More than just a matter of life and death. Nat. Rev. Cancer.

[2] Adhikary S, Eilers M (2005). Transcriptional regulation and transformation by Myc proteins. Nat. Rev. Mol. Cell Biol..

[3] Gabay M, Li Y, Felsher DW (2014). MYC activation is a hallmark of cancer initiation and maintenance. Cold Spring Harb. Perspect. Med..

[4] Lancho O, Herranz D (2018). The MYC Enhancer-ome: Long-Range Transcriptional Regulation of MYC in Cancer. Trends Cancer.

[5] Ahmadiyeh N (2010). 8q24 prostate, breast, and colon cancer risk loci show tissue-specific long-range interaction with MYC. Proc. Natl. Acad. Sci. U S A.

[6] Hnisz D (2013). Super-enhancers in the control of cell identity and disease. Cell.

[7] Oktay Y (2016). IDH-mutant glioma specific association of rs55705857 located at 8q24.21 involves MYC deregulation. Sci. Rep..

[8] Balogh J (2016). Hepatocellular carcinoma: A review. J. Hepatocell. Carcinoma.

[9] Yang Y (2019). The chromatin remodeling protein BRG1 links ELOVL3 trans-activation to prostate cancer metastasis. Biochim. Biophys. Acta.

[10] Liu M, Jiang L, Guan XY (2014). The genetic and epigenetic alterations in human hepatocellular carcinoma: A recent update. Protein Cell.

[11] Zender L (2010). Cancer gene discovery in hepatocellular carcinoma. J. Hepatol..

[12] Zheng K, Cubero FJ, Nevzorova YA (2017). c-MYC-making liver sick: Role of c-MYC in hepatic cell function, homeostasis disease. Genes.

[13] Kalkat M (2017). MYC deregulation in primary human cancers. Genes.

[14] Hsieh CL (2014). Enhancer RNAs participate in androgen receptor-driven looping that selectively enhances gene activation. Proc. Natl. Acad. Sci. USA.

[15] Arnold PR, Wells AD, Li XC (2019). Diversity and emerging roles of enhancer RNA in regulation of gene expression and cell fate. Front. Cell Dev. Biol..

[16] Lee JH, Xiong F, Li W (2020). Enhancer RNAs in cancer: Regulation, mechanisms and therapeutic potential. RNA Biol..

[17] Bhagwat AS (2016). BET bromodomain inhibition releases the mediator complex from select cis-regulatory elements. Cell Rep..

[18] Donati B, Lorenzini E, Ciarrocchi A (2018). BRD4 and cancer: Going beyond transcriptional regulation. Mol. Cancer.

[19] Kanno T (2014). BRD4 assists elongation of both coding and enhancer RNAs by interacting with acetylated histones. Nat. Struct. Mol. Biol..

[20] Bid HK (2016). The Bromodomain BET inhibitor JQ1 suppresses tumor angiogenesis in models of childhood sarcoma. Mol. Cancer Ther..

[21] Goel HL, Mercurio AM (2013). VEGF targets the tumour cell. Nat. Rev. Cancer.

[22] Kaseb AO (2009). Vascular endothelial growth factor in the management of hepatocellular carcinoma: A review of literature. Cancer.

[23] Benhammou JN (2019). Novel lipid long intervening noncoding RNA, oligodendrocyte maturation-associated long intergenic noncoding RNA, regulates the liver steatosis gene stearoyl-coenzyme A desaturase as an enhancer RNA. Hepatol. Commun..

[24] Bouvy-Liivrand M (2017). Analysis of primary microRNA loci from nascent transcriptomes reveals regulatory domains governed by chromatin architecture. Nucleic Acids Res..

[25] Consortium, E. P. (2012). An integrated encyclopedia of DNA elements in the human genome. Nature.

[26] Gertz J (2013). Distinct properties of cell-type-specific and shared transcription factor binding sites. Mol. Cell.

[27] Viiri LE (2019). Extensive reprogramming of the nascent transcriptome during iPSC to hepatocyte differentiation. Sci. Rep..

[28] Bernstein BE (2010). The NIH roadmap epigenomics mapping consortium. Nat. Biotechnol..

[29] Zhang X (2016). Identification of focally amplified lineage-specific super-enhancers in human epithelial cancers. Nat. Genet..

[30] Zhu HQ (2015). Aberrant expression of CCAT1 regulated by c-Myc predicts the prognosis of hepatocellular carcinoma. Asian Pac. J. Cancer Prev..

[31] Wang Y (2018). The 3D Genome Browser: a web-based browser for visualizing 3D genome organization and long-range chromatin interactions. Genome Biol..

[32] Fernandez PC (2003). Genomic targets of the human c-Myc protein. Genes Dev.

[33] Khattar E, Tergaonkar V (2018). Transcriptional regulation of telomerase reverse trnascriptase (TERT) by MYC. J. Hepatol..

[34] Dave K (2017). Mice deficient of Myc super-enhancer region reveal differential control mechanism between normal and pathological growth. Elife.

[35] Tak YG (2016). Effects on the transcriptome upon deletion of a distal element cannot be predicted by the size of the H3K27Ac peak in human cells. Nucleic Acids Res..

[36] Florea V (2013). c-MYC is Essential to prevent endothelial pro-inflammatory senescent phenotype. PLoS ONE.

[37] Kim TK (2010). Widespread transcription at neuronal activity-regulated enhancers. Nature.

[38] Sur I, Taipale J (2016). The role of enhancers in cancer. Nat. Rev. Cancer.

[39] Loven J (2013). Selective inhibition of tumor oncogenes by disruption of super-enhancers. Cell.

[40] Shi J, Vakoc CR (2014). The mechanisms behind the therapeutic activity of BET bromodomain inhibition. Mol. Cell.

[41] Pomerantz MM (2015). The androgen receptor cistrome is extensively reprogrammed in human prostate tumorigenesis. Nat. Genet..

[42] Andersson R (2014). An atlas of active enhancers across human cell types and tissues. Nature.

[43] Park A (2020). Global epigenomic analysis of KSHV-infected primary effusion lymphoma identifies functional MYC superenhancers and enhancer RNAs. Proc. Natl. Acad. Sci. U S A.

[44] Schuijers J (2018). Transcriptional dysregulation of MYC reveals common enhancer-docking mechanism. Cell Rep.

[45] Aden D (1979). Controlled synthesis of HBsAh in a differentiated human liver carcinoma-derived cell line. Nature.

[46] Nakabayashi H (1982). Growth of human hepatoma cells lines with differentiated functions in chemically defined medium. Cancer Res..

[47] Kaczkowski B (2016). Transcriptome analysis of recurrently deregulated genes across multiple cancers identifies new pan-cancer biomarkers. Cancer Res..

[48] Oh S (2021). Enhancer release and retargeting activates disease-susceptibility genes. Nature.

[49] Pnueli L, Rudnizky S, Yosefzon Y, Melamed P (2015). RNA transcribed from a distal enhancer is required for activating the chromatin at the promoter of the gonadotropin alpha-subunit gene. Proc. Natl. Acad. Sci. USA.

[50] Pan CW (2021). Functional roles of antisense enhancer RNA for promoting prostate cancer progression. Theranostics.

[51] Zhang Z (2019). Transcriptional landscape and clinical utility of enhancer RNAs for eRNA-targeted therapy in cancer. Nat. Commun..

[52] Jiao W (2018). HPSE enhancer RNA promotes cancer progression through driving chromatin looping and regulating hnRNPU/p300/EGR1/HPSE axis. Oncogene.

[53] Liang J (2016). Epstein-Barr virus super-enhancer eRNAs are essential for MYC oncogene expression and lymphoblast proliferation. Proc. Natl. Acad. Sci. USA.

[54] Zhang HL, Wang P, Lu MZ, Zhang SD, Zheng L (2019). c-Myc maintains the self-renewal and chemoresistance properties of colon cancer stem cells. Oncol. Lett..

[55] Delmore JE (2011). BET bromodomain inhibition as a therapeutic strategy to target c-Myc. Cell.

[56] Li GQ (2016). Suppression of BRD4 inhibits human hepatocellular carcinoma by repressing MYC and enhancing BIM expression. Oncotarget.

[57] Sengupta D (2015). Disruption of BRD4 at H3K27Ac-enriched enhancer region correlates with decreased c-Myc expression in Merkel cell carcinoma. Epigenetics.

[58] Kang SC (2015). Transcriptomic profiling and H3K27me3 distribution reveal both demethylase-dependent and independent regulation of developmental gene transcription in cell differentiation. PLoS ONE.

[59] Bolger AM, Lohse M, Usadel B (2014). Trimmomatic: A flexible trimmer for Illumina sequence data. Bioinformatics.

[60] Langmead B, Salzberg SL (2012). Fast gapped-read alignment with Bowtie 2. Nat. Methods.

[61] Dobin A (2013). STAR: Ultrafast universal RNA-seq aligner. Bioinformatics.

[62] Heinz S (2010). Simple combinations of lineage-determining transcription factors prime cis-regulatory elements required for macrophage and b cell identities. Mol. Cell.

[63] Mali P (2013). RNA-guided human genome engineering via Cas9. Science.

