# Supplementary material for: Targeting of non-coding RNAs encoded by novel MYC enhancers inhibits the proliferation of human hepatic carcinoma cells in vitro
Source: Sci Rep. 2022 Jan 17;12:855. doi: 10.1038/s41598-022-04869-w (PMC8764030; doi:10.1038/s41598-022-04869-w)
Supplement: Supplementary file 1 — Supplementary Information. [file 41598_2022_4869_MOESM1_ESM.docx]

**Targeting of noncoding RNAs encoded by a novel *MYC* enhancer inhibits the proliferation of human hepatic carcinoma cells in vitro**

Hae In Choi^1^, Ga Yeong An^1^, Eun Yeoung Yoo^1^, Mina Baek^2,3^, Jin Choul Chai^4^, Bert Binas^2^, Young Seek Lee^4^, Kyoung Hwa Jung^5*^, Young Gyu Chai^1,2*^

^1^ Department of Bionanotechnology, Hanyang University, Seoul, 04673, Republic of Korea.

^2^ Department of Molecular & Life Science, Hanyang University, Ansan, 15588, Republic of Korea.

^3^ Institute of Natural Science and Technology, Hanyang University, Ansan, 15588, Republic of Korea.

^4^ College of Veterinary Medicine, Seoul National University, Seoul, 08826, Republic of Korea.

^5^Convergence Technology Campus of Korea Polytechnic II, Incheon, 21417, Republic of Korea.

* Corresponding authors

**Supplementary figures**

**Figure S1**

**
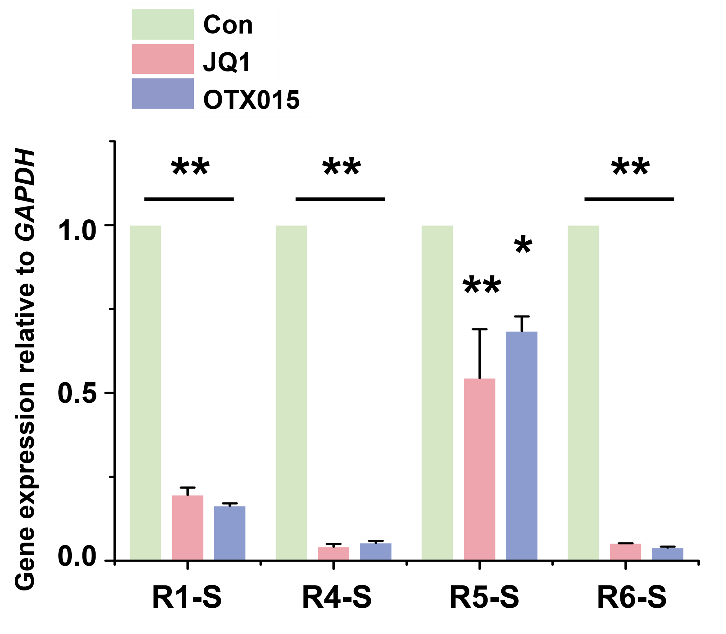
**

**Figure S1. eRNA expression of *MYC* enhancer regions in HepG2 cells**

qRT-PCR of eRNA transcription levels in BET inhibitor-treated HepG2 cells (5 µM, 24 h). The values are the mean ± SD from triplicate well measurements. *, *p* < 0.05 and **, *p* < 0.01.

**Figure S2**

**
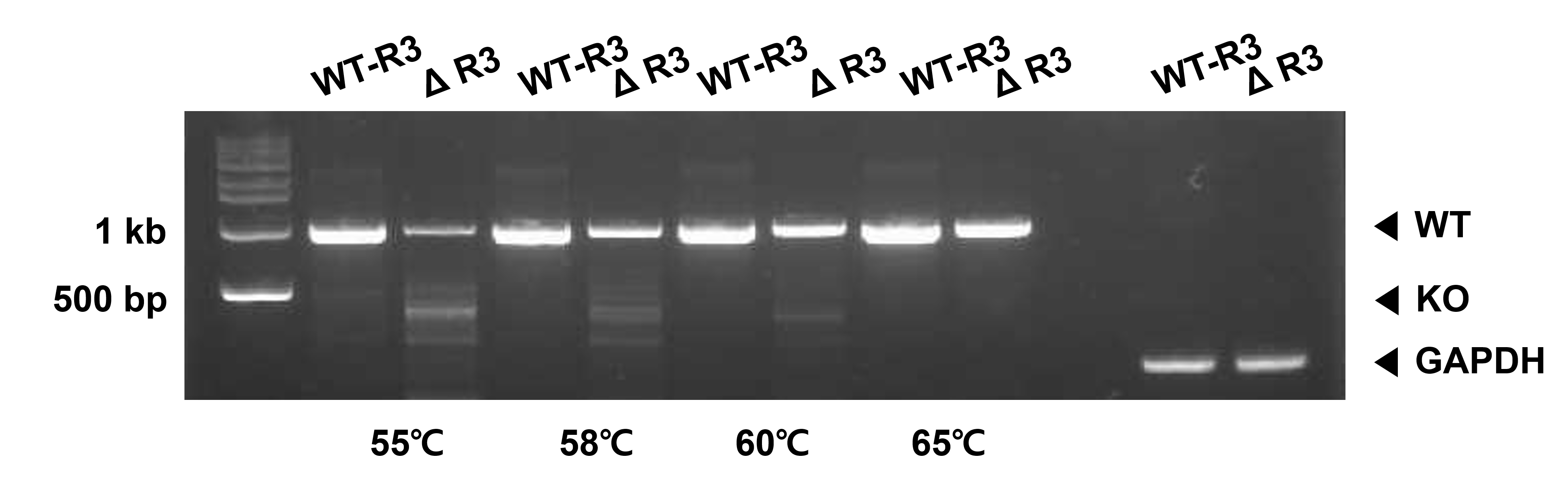
**

**Figure S2. Deletion of R3 in Huh7 cells**

Agarose gel electrophoresis of the PCR products that identify the WT vs. knockout (KO) alleles (Primer information in Table 3). A deletion of 357 bp of the R3 region was confirmed in the band with a size of about 500 bp.

**Supplementary Tables**

**Table S1. Primer sequences used for qRT-PCR**

| **Gene** | **Forward (5’-3’)** | **Reverse (5’-3’)** |
| --- | --- | --- |
| GAPDH | AAGGTCGGAGTCAACGGATT | CTCCTGGAAGATGGTGATGG |
| MYC | GGCTCCTGGCAAAAGGTCA | AGTTGTGCTGATGTGTGGAGA |
| DUSP9 | CAGCCGTTCTGTCACCGTC | CAAGCTGCGCTCAAAGTCC |
| VEGFA | TTATGCGGATCAAACCTCACC | GAAGCTCATCTCTCCTATGTGC |
| PVT1 | GCCCCTTCTATGGGAATCACTA | GGGGCAGAGATGAAATCGTAAT |
| CCAT1 | GGGCTGGTACAGACTAGGGA | TAAGCAGGTCAGAAAGGGCG |
| FAM49B | CTCAAGATGACAAATCCTGC | CCGGTACATTGTTAATCCTC |
| ICAM1 | ATGCCCAGACATCTGTGTCC | GGGGTCTCTATGCCCAACAA |
| IRF2 | AATGCTGCCCCTATCAGAACG | CAGGACCGCATACTCAGGAGA |
| TERT | AAATGCGGCCCCTGTTTCT | CAGTGCGTCTTGAGGAGCA |
| **eRNA** | **Forward (5’-3’)** | **Reverse (5’-3’)** |
| R1 | GGCTGGAGCCAGAAAGCTAA | GTCTGTGGCTCAGATGCTGT |
| R2 | ACTGCAGCCACACTTCTGTT | TCTTGCTGCAATCCAGAGGG |
| R3 | GCACTTGTTCCTACCTCCGC | ATGTAGTGGGACCATCACCC |
| R4 | ATGCCATTTTGGGCCTTCAATAAG | TGCTACAAGTATGTGCTGACC |
| R5 | GCCAGGGATGAAAAGGCCAA | TCACAGTGTAGGGGGTGTGT |
| R6 | AGGTGCATTTAGCCCCAGTC | GTCTCTGGGTTCCAAGCTCC |

**Table S2. Primers for cloning**

| **Enhancer** | **Sequence (5’-3’)** |
| --- | --- |
| LUAD-R3-F_NheⅠ | ATAGCTAGCTGCCAATGGTCAGTTCTCTG |
| LUAD-R3-R_XhoⅠ | ATACTCGAGGTCACCATTGGTACCCCAAG |
| LUAD-R4-F_SacⅠ | ATAGAGCTCTAGCCTACAGGGACCAATGC |
| LUAD-R4-R_XhoⅠ | ATACTCGAGCAGCAGTGGACAAAACCAAA |
| R1-F_NheI | ATAGCTAGCGCGTTTCCTGAAACACCACC |
| R1-R_XhoI | ATACTCGAGAGAGGAAGGCCATGCCTAGA |
| R2-F_NheI | ATAGCTAGCAGGTTAATGCAGAGCCTCCTC |
| R2-R_XhoI | ATACTCGAGTGAAAAGTCAAGTGCCGTCTG |
| R3-F_NheI | ATAGCTAGCCCCTTTCAAGGGCAAAGACGA |
| R3-R_XhoI | ATACTCGAGTGTCCTGAGAGTGGAGGCTTA |
| R4-F_NheI | ATAGCTAGCCGTCTCAGTTTCCAGAGGGTTC |
| R4-R_XhoI | ATACTCGAGTCTTAGATTGAGACAGGGAGTTGA |
| R5-F_NheI | ATAGCTAGCTAGGTTGCCTGCCCTAGTAGT |
| R5-R_XhoI | ATACTCGAGATTCTACCCGGTGTACAGCAC |
| R6-F_NheI | ATAGCTAGCCTTTCTGCCAGGCAACATGC |
| R6-R_XhoI | ATACTCGAGTGACCACTCCATGCCAGTTC |
| R2-1-F_NheI | ATAGCTAGCAGGTTAATGCAGAGCCTCCTC |
| R2-1_R_XhoI | ATACTCGAGTTAGATGAGGAAACCGAGGCAT |
| R2-2-F_NheI | ATAGCTAGCATCTGATTTCTATGAAGCTTTGAC |
| R2-2-R_XhoI | ATACTCGAGCAATTTCCGAACGCTTGAGA |
| R2-3-F_NheI | ATAGCTAGCTCCTTTCTCAAGCGTTCGGA |
| R2-3-R_XhoI | ATACTCGAGTGTCGGCAAGCTCAAAATGC |
| R2-4-F_NheI | ATAGCTAGCTTTAGAGCTCAAGTGCGCCA |
| R2-4-R_XhoI | ATACTCGAGTGTGGCTCTTGCCTTCAGAG |
| R2-5-F_NheI | ATAGCTAGCCTTGCAGGCTGCTCTCTTTC |
| R2-5-R_XhoI | ATACTCGAGTGAAAAGTCAAGTGCCGTCTG |
| R3-1-F_NheI | ATAGCTAGCCCCTTTCAAGGGCAAAGACGA |
| R3-1-R_XhoI | ATACTCGAGCTGAACCCAGAAATATGAAGGAC |
| R3-2-F_NheI | ATAGCTAGCTGGGTTTCTAGGTTTTAGTCCTTC |
| R3-2-R_XhoI | ATACTCGAGCGTGTCATTACAGTGCAGAGG |
| R3-3-F_NheI | ATAGCTAGCCCTCTGCACTGTAATGACACGA |
| R3-3-R_XhoI | ATACTCGAGGCCTGGCAAACTTGTTTTGAGAA |
| R3-4-F_NheI | ATAGCTAGCTAGGCACTTTCAGCTGGTCA |
| R3-4-R_XhoI | ATACTCGAGTGTCCTGAGAGTGGAGGCTTA |

**Table S3. ASO and sgRNA target sequence.**

| **ASO** | **Sequence (5’-3’)** |
| --- | --- |
| ASO-R2 | CCTTCAAAGCATCACG |
| ASO-R3 | CTAGTTTCCACTGACA |
| **sgRNA** | **Sequence (5’-3’)** |
| MYC-R3-gRNA-1 | AACTGAACCCAGAAATATGA |
| MYC-R3-gRNA-2 | CTCGTGTCATTACAGTGCAG |
| **Deletion confirm primer** | **Sequence (5’-3’)** |
| MYC-E3-Del-F | ATTTGGCAACTCAGACGGCA |
| MYC-E3-Del-R | CACCACTGCTTCCTCTCACC |

**Table S4. MYC enhancer regions**

| **Enhancer** | **h38_DNA range** |
| --- | --- |
| LUAD-R3 | chr8:128176171-128177714 |
| LUAD-R4 | chr8:128183039-128183988 |
| R1 | chr8:128184800-128186225 |
| R2 | chr8:128554145-128555945 |
| R3 | chr8:128556059-128557653 |
| R4 | chr8:128582625-128583855 |
| R5 | chr8:128584574-128585781 |
| R6 | chr8:128601617-128603273 |
